# Assembly of a dsRNA synthesizing complex: RNA-DEPENDENT RNA POLYMERASE 2 contacts the largest subunit of NUCLEAR RNA POLYMERASE IV

**DOI:** 10.1073/pnas.2019276118

**Published:** 2021-03-22

**Authors:** Vibhor Mishra, Jasleen Singh, Feng Wang, Yixiang Zhang, Akihito Fukudome, Jonathan C. Trinidad, Yuichiro Takagi, Craig S. Pikaard

**Affiliations:** ^a^HHMI, Indiana University, Bloomington, IN 47405;; ^b^Department of Biology and Department of Molecular and Cellular Biochemistry, Indiana University, Bloomington, IN 47405;; ^c^Department of Chemistry, Indiana University, Bloomington, IN 47405;; ^d^Laboratory for Biological Mass Spectrometry, Indiana University, Bloomington, IN 47405;; ^e^Department of Biochemistry and Molecular Biology, Indiana University School of Medicine, Indianapolis, IN 46202

**Keywords:** RNA-directed DNA methylation, gene silencing, Pol IV, RDR2, RNA interference

## Abstract

In plants, transcription of selfish genetic elements such as transposons and DNA viruses is suppressed by RNA-directed DNA methylation. This process is guided by 24-nt short-interfering RNAs (siRNAs) whose double-stranded precursors are synthesized by DNA-dependent NUCLEAR RNA POLYMERASE IV (Pol IV) and RNA-DEPENDENT RNA POLYMERASE 2 (RDR2). Pol IV and RDR2 coimmunoprecipitate, and their activities are tightly coupled, yet the basis for their association is unknown. Here, we show that an interval near the RDR2 active site contacts the Pol IV catalytic subunit, NRPD1, the largest of Pol IV’s 12 subunits. Contacts between the catalytic regions of the two enzymes suggests that RDR2 is positioned to rapidly engage the free 3′ ends of Pol IV transcripts and convert these single-stranded transcripts into double-stranded RNAs (dsRNAs).

## Significance

Short interfering RNAs (siRNAs) can inhibit mRNA translation or guide chromatin modifications that inhibit transcription, thus impacting gene regulation. In plants, transcriptional gene silencing involves siRNAs whose double-stranded (ds) precursors are generated by the coupled activities of NUCLEAR RNA POLYMERASE IV and RNA-DEPENDENT RNA POLYMERASE 2. We present evidence that Pol IV-RDR2 association involves contact between RDR2 and NRPD1, Pol IV’s largest catalytic subunit. As the only subunit never shared by Pol II or Pol IV, NRPD1 interaction accounts for RDR2's specific association with Pol IV. The positions of the protein docking sites suggest that Pol IV transcripts are generated in close proximity to RDR2’s catalytic site, enabling RDR2 to efficiently engage Pol IV transcripts and convert them into dsRNAs.

In eukaryotes, noncoding RNAs guide transcriptional gene silencing to keep transposons, repeated elements, and viruses in check, thus defending against genome instability (1–3). The required noncoding RNAs include short interfering RNAs (siRNAs) and longer scaffold RNAs to which siRNAs, associated with an Argonaute family protein, base pair to facilitate the recruitment of chromatin modifying complexes to the associated chromosomal locus (4–6). In most eukaryotes, noncoding silencing RNAs are synthesized by DNA-dependent RNA Polymerase II (Pol II). However, plants evolved from Pol II two DNA-dependent RNA polymerases, NUCLEAR RNA POLYMERASE IV (Pol IV) and NUCLEAR RNA POLYMERASE V (Pol V) (7), that specialize in noncoding RNA synthesis and play nonredundant roles in a process known as RNA-directed DNA methylation (8, 9). Pol IV transcribes chromosomal DNA to generate relatively short transcripts of 25–45 nt (10, 11). An RNA-dependent RNA polymerase, RDR2 (12) then uses the Pol IV transcripts as templates to synthesize complementary strands, yielding double-stranded RNAs (dsRNAs) (13). Resulting dsRNAs are then cleaved by DICER-LIKE 3 (DCL3) to produce siRNAs that can be 24 nt or 23 nt in length (13). The 24-nt siRNAs become stably associated with ARGONAUTE 4 (AGO4), or related Argonaute family members, yielding RNA-induced silencing complexes (RISCs) (14–16). RISCs then find their target loci via siRNA basepairing with long noncoding scaffold RNAs transcribed by NUCLEAR RNA POLYMERASE V (Pol V) (17, 18) and by protein–protein interactions between AGO4 and the C-terminal domain (CTD) of the Pol V largest subunit (19) or between AGO4 and the Pol V-associated protein, SPT5L (20, 21). Once localized at target loci, RISCs facilitate recruitment of the de novo DNA methyltransferase, DRM2 (DOMAINS REARRANGED METHYLTRANSFERASE 2) (22) and histone-modifying activities that act in coordination with DNA methylation (8, 9, 23). Collectively, these activities generate chromatin states that are refractive to promoter-dependent gene transcription, thus accounting for gene silencing.

Using purified Pol IV, RDR2, and DCL3, we recently recapitulated the siRNA biogenesis phase of the RNA-directed DNA methylation pathway in vitro (13). These experiments revealed that Pol IV and RDR2 enzymatic activities are coupled, with Pol IV’s distinctive termination mechanism facilitating the direct handoff, or channeling, of transcripts to RDR2 (24). In keeping with their coordinated activities, Pol IV and RDR2 coimmunoprecipitate in *Arabidopsis thaliana* (25, 26) and *Zea mays* (in maize, the RDR2 ortholog is MOP1) (27). However, it is not known whether the two enzymes interact directly or associate via one or more bridging molecules.

Using several independent methods, we show that recombinant RDR2 can directly interact with the largest catalytic subunit of Pol IV, NRPD1. Using deletion constructs, synthetic peptide arrays, chemical cross-linking, and mass spectroscopy, we identify sequences in the amino-terminal region of NRPD1 that contributes to the folding of the catalytic center that interact with RDR2 near its catalytic center. These results suggest that RDR2–NRPD1 interaction brings the active sites of the two enzymes into close proximity, enabling RDR2 to efficiently engage the 3′ ends of terminated Pol IV transcripts and convert them into dsRNAs.

## Results

### Recombinant RDR2 has RNA-Dependent RNA Polymerase Activity.

**Fig. 1.**
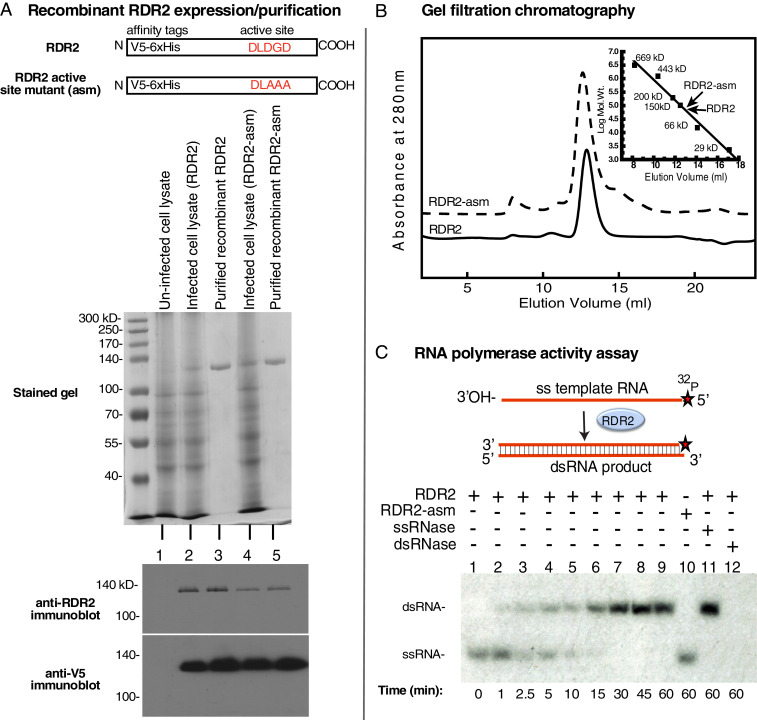
Purification and activity of recombinant RDR2. (*A*) The cartoons depict constructs for WT RDR2 or an active site mutant (asm) that has amino acids of the magnesium binding site changed to alanines. Both proteins have N-terminal V5 epitope tags and 6× His tags. The stained SDS-PAGE gel shows lysates of uninfected High Five insect cells, cells infected with the RDR2 or RDR2-asm baculovirus vectors, and the purified proteins. Anti-RDR2 or anti-V5 immunoblots of the samples are shown at the bottom. (*B*) Gel filtration chromatography profiles of recombinant RDR2 (solid line) and recombinant RDR2-asm (dashed line). *Inset* shows a semilog plot comparing elution volumes of protein mass standards, RDR2 and RDR2-asm. (*C*) Conversion of a 37-nt ssRNA, labeled on its 5′ end with ^32^P, into dsRNA by recombinant RDR2. Lanes 1–9, time-course of conversion of ssRNA into dsRNA. Lane 10, catalytically dead RDR2-asm was substituted for WT RDR2. Lanes 11 and 12, reaction products treated with a ssRNA-specific RNase, RNase ONE, or dsRNA-specific RNase, RNase V1. Reaction products were resolved on a 15% polyacrylamide native gel and visualized by autoradiography.

Using a baculovirus vector, we expressed in insect cells a recombinant gene that encodes WT RDR2 engineered to have both a V5 epitope tag and 6xHis tag at the N terminus. A derivative active site mutant (RDR2-asm; Fig. 1*A*) was similarly expressed. Nickel affinity and gel filtration chromatography yielded highly purified RDR2 proteins (Fig. 1*A*, lanes 3 and 5) whose identities were verified by immunoblotting using both anti-V5 and anti-RDR2 antibodies (Fig. 1 *A*, *Bottom*). Upon analytical gel filtration chromatography, the WT and mutant forms of RDR2 elute as single peaks with estimated masses of ∼140 kDa (Fig. 1 *B*, *Inset*), in good agreement with their predicted monomeric masses of ∼134 kDa.

To test for RNA-dependent RNA polymerase activity, RDR2 and RDR2-asm were provided a 37-nt single-stranded (ss) RNA template labeled with ^32^P on its 5′ end and all four nucleoside triphosphates. Conversion of ssRNA into dsRNA was then monitored by native gel electrophoresis and autoradiography (Fig. 1*C*). Using RDR2, dsRNA products are detected within 1 min. In contrast, the RDR2-asm mutant showed no detectable activity, even after 60 min (lane 10). Reaction products of RDR2 are resistant to the ssRNA-specific ribonuclease, RNase ONE, but are sensitive to the dsRNA-specific ribonuclease, RNase V1 (see lanes 11 and 12). Collectively, these results show that recombinant RDR2 can catalyze dsRNA synthesis using an ssRNA template, in vitro, with second-strand synthesis beginning at, or very near, the 3′ terminus of the RNA template.

### The Pol IV-RDR2 Complex can be Reconstituted Using Recombinant RDR2.

**Fig. 2.**
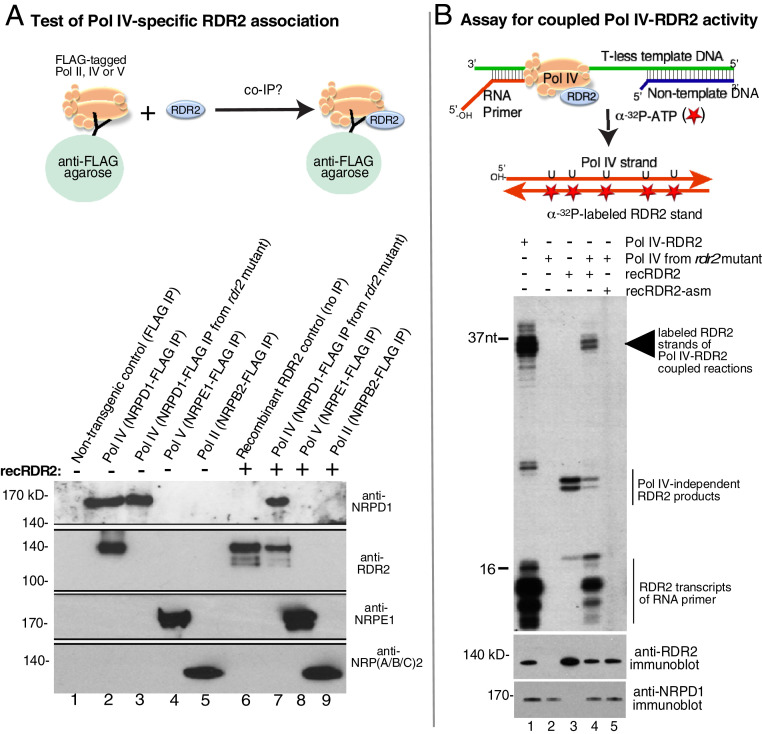
Reconstitution of a functional Pol IV-RDR2 complex. (*A*) Recombinant RDR2 stably associates with Pol IV. Pol IV, Pol V, and Pol II assembled using FLAG-tagged NRPD1, NRPE1, or NRPB2, respectively, were IPed from transgenic plants using anti-FLAG resin. In lane 1, anti-FLAG IP of a nontransgenic plant lysate serves as a negative control. In lane 2, Pol IV was IPed from plants that are wild type for RDR2. In lanes 3 and 7, Pol IV IPed from a *rdr2* null mutant. In lanes 7–9, recombinant RDR2 was mixed with the indicated Pol IV, Pol V, or Pol II fractions prior to anti-FLAG IP. Lane 6 shows recombinant RDR2 as a positive control. Proteins resolved by SDS-PAGE were subjected to immunoblotting using anti-RDR2, anti-NRPD1, anti-NRPE1, or an antibody that recognizes a peptide sequence of the Pol II subunit, NRPB2 that is also present in the NRPA2 and NRPC2 subunits of Pols I and III, respectively. (*B*) Pol IV-RDR2 assembled using recombinant RDR2 carries out the coupled Pol IV-RDR2 reaction, generating dsRNA from a DNA template. The cartoon illustrates the use of a T-less DNA template and RNA labeling with α-[^32^P]ATP to specifically label second RNA strands synthesized by RDR2 (13). Lane 1 shows the activity of Pol IV-RDR2 complexes isolated from plants expressing WT RDR2. Lane 2 tests the activity of Pol IV purified from the *rdr2* null mutant background. Lane 3 tests the activity of recombinant RDR2 alone. In lanes 4 and 5, Pol IV-RDR2 complexes were reconstituted using recombinant RDR2 or catalytically dead RDR2-asm and tested for activity. The bottom image shows immunoblots to detect NRPD1 or RDR2 present in the reactions.

Pol IV and RDR2 copurify and coimmunoprecipitate (co-IP) in both *A. thaliana* and maize (25–27). We tested whether Pol IV-RDR2 association can be recapitulated in vitro using recombinant RDR2 (Fig. 2). For this experiment, we made use of an *A. thaliana* transgenic line in which NRPD1 bearing a FLAG epitope tag at its C terminus (NRPD1-FLAG) rescues an *nrpd1-3* null mutant (28). This allows Pol IV to be affinity-purified using anti-FLAG resin (see cartoon of Fig. 2*A*). Using polyclonal antibodies recognizing native NRPD1 or native RDR2, both proteins are detected upon NRPD1-FLAG IP (Fig. 2*A* lane 2), in agreement with prior studies (26). If NRPD1-FLAG immunoprecipitation (IP) is used to affinity-capture Pol IV from a *rdr2* null mutant background, RDR2 is not detected, as expected (lane 3). RDR2 is also not detected in Pol V or Pol II fractions affinity-purified by virtue of FLAG-tagged NRPE1 (the largest subunit of Pol V) or NRPB2, the second-largest subunit of Pol II (lanes 4 and 5), in keeping with RDR2’s specific association with Pol IV (28).

Pol IV, Pol V, and Pol II were next immunoprecipitated (IPed) following incubation with recombinant RDR2 (Fig. 2*A*, lanes 7–9). Importantly, Pol IV purified from the *rdr2* null mutant interacts with recombinant RDR2 such that both proteins co-IP (Fig. 2*A*, lane 7). By contrast, Pols II or V do not pull down RDR2 (lanes 8 and 9).

We next tested whether the Pol IV-RDR2 complexes assembled using recombinant RDR2 can carry out the coupled transcription reactions that generate dsRNA from a DNA template strand, as demonstrated by Singh et al. (13). In this assay, diagrammed in Fig. 2*B*, Pol IV transcription is initiated using an RNA primer hybridized to a T-less (no thymidines) 51-nt DNA template strand. Importantly, Pol IV transcription does not require a RNA primer, but use of a primer ensures that the Pol IV transcripts have a defined 5′ end, rather than alternative start sites. When the elongating Pol IV encounters a 28-nt nontemplate strand of DNA, basepaired to the template strand, Pol IV transcribes only ∼12–16 nt into the double-stranded region and then terminates, yielding transcripts of ∼34–37 nt (13). Importantly, Pol IV termination induced by the basepaired nontemplate strand is required for RDR2 to engage the Pol IV transcript and synthesize the complementary RNA strand (13). Because no thymidines are present in the DNA template, α-[^32^P]ATP is not incorporated into the initial Pol IV transcripts. However, uracils incorporated into the Pol IV transcripts specify the incorporation of α-[^32^P]ATP into the RNA second strands synthesized by RDR2. Thus α-[^32^P]ATP incorporation into RNAs of ∼34–37 nt is indicative of second-strand RNA synthesis and coupled Pol IV-RDR2 transcription (13).

Lane 1 of Fig. 2*B* shows reaction products generated by Pol IV associated with WT RDR2 (see immunoblots at bottom), with prominent 34- to 37-nt body-labeled RDR2 transcripts readily apparent. Pol IV purified from a *rdr2* null mutant does not generate these transcripts (lane 2). The ladder of short RNA transcripts near the bottom of lane 1 are RDR2 transcripts generated using the 16-nt RNA primer as a template (26). Haag et al. showed that RDR2 associated with Pol IV generates these short primer transcripts but RDR2 isolated from a *pol iv* mutant does not (26). In keeping with these observations, recombinant RDR2 does not generate the short primer transcripts (Fig. 2*B*, lane 3), although longer transcripts of ∼22–23 nt are observed. Adding recombinant RDR2 to the Pol IV fraction reconstitutes the Pol IV-RDR2–coupled reaction (lane 4), generating 34- to 37-nt transcripts like those generated by native Pol IV-RDR2 (compare lanes 4 and 1). Short RDR2 transcripts derived from the RNA primer are also observed for the reconstituted Pol IV-RDR2 complex (lane 4). Importantly, no products are observed in reactions in which Pol IV-RDR2 complexes were reconstituted using RDR2 mutated at its active site (RDR2-asm; lane 5).

### RDR2 Physically Interacts with the Largest Catalytic Subunit of Pol IV.

**Fig. 3.**
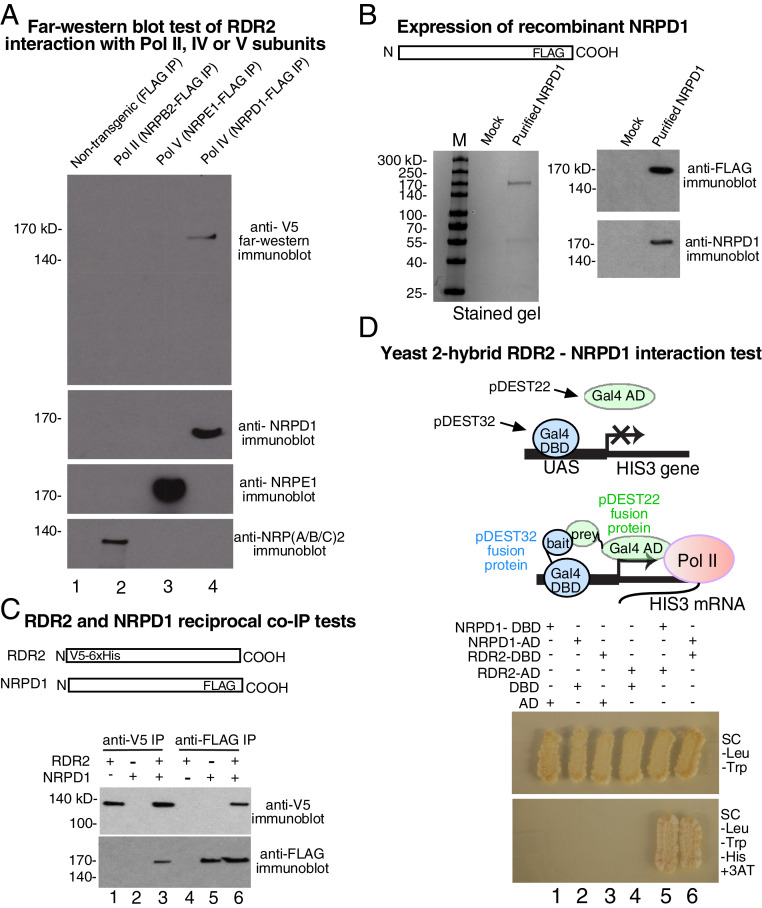
RDR2 interacts with the Pol IV largest subunit, NRPD1. (*A*) Far-Western blot test for RDR2 interacting proteins. Pols II, V, and IV assembled using FLAG-tagged NRPB2, NRPE1, or NRPD1, respectively, were IPed using anti-FLAG resin. Following SDS-PAGE and electroblotting, the filter was incubated with recombinant V5-tagged RDR2. After washing, the filter was incubated with anti-V5 antibody to detect immobilized RDR2. Immunoblots at the bottom of the image detect NRPD1 (Pol IV), NRPE1 (Pol V), or NRPB2. (*B*) Expression of recombinant NRPD1. Shown at left is Coomassie-stained SDS-PAGE comparing purified recombinant NRPD1 expressed in High Five insect cells to mock-infected cells subjected to the same purification protocol. At right are anti-FLAG and anti-NRPD1 immunoblots of these fractions. (*C*) Recombinant NRPD1 and recombinant RDR2 coimmunoprecipitate. V5-tagged RDR2 and FLAG-tagged NRPD1 were incubated alone or together, as indicated, then IPed using anti-V5 or anti-FLAG resins. IPed fractions were then subjected to SDS-PAGE and immunoblotting using anti-V5 or anti-FLAG antibodies. (*D*) NRPD1 and RDR2 interact in reciprocal yeast two-hybrid interaction tests. The cartoon depicts the experiment, using proteins fused to the Gal4 DNA binding domain (DBD) as bait and proteins fused to the Gal4 activation domain (AD) as prey. Upon cotransformation of the fusion constructs into yeast, the yeast are able to grow on media lacking tryptophan and leucine (*Upper*). Interaction between the indicated fusion proteins is demonstrated by growth on media lacking tryptophan, leucine, histidine, and with addition of 3AT (Lower). UAS, upstream activating sequence that GAL4 binds.

Having demonstrated that recombinant RDR2 can associate with Pol IV to form a functional complex capable of dsRNA synthesis, we sought to identify the basis for Pol IV-RDR2 association. As an initial test, we performed a far-Western blot in which FLAG-tagged Pol II, Pol IV, and Pol V were affinity-purified, subjected to sodium dodecyl sulfate polyacrylamide gel electrophoresis (SDS-PAGE), electroblotted to nitrocellulose, and incubated with recombinant V5-tagged RDR2. Filters were washed extensively then incubated with anti-V5 antibody conjugated to horseradish peroxidase (HRP). Development of the blot by chemiluminescent detection of HRP activity revealed a single band of ∼165 kDa in the Pol IV fraction, consistent with the size of the largest Pol IV subunit, NRPD1 (Fig. 3*A*, lane 4).

To test whether RDR2 can interact with NRPD1, we designed a synthetic transgene encoding NRPD1 fused at its C terminus to a FLAG epitope tag, expressed the protein in insect cells using a baculovirus vector, and purified the protein to near homogeneity (Fig. 3*B*). Upon incubation with V5-tagged RDR2, the proteins co-IP using either anti-V5 or anti-FLAG resin, indicating that NRPD1 and RDR2 can, indeed, interact (Fig. 3*C*, lanes 3 and 6).

As a third test of RDR2’s ability to interact with NRPD1, we performed yeast two-hybrid interaction experiments (Fig. 3*D*) using NRPD1 or RDR2 as both bait (when fused to the Gal4 DNA binding domain, DBD of pDEST32) and prey (when fused to the Gal4 activation domain, AD of pDEST22). Each combination of NRPD1 and RDR2, as bait or prey, allowed their interaction to facilitate HIS3 expression, enabling colony growth on media lacking histidine and containing 25 mM 3 amino-1,2,3-triazole (3AT) to increase the stringency of HIS selection (Fig. 3*D*, lanes 5 and 6).

### The N-Terminal Region of NRPD1 Interacts with RDR2.

**Fig. 4.**
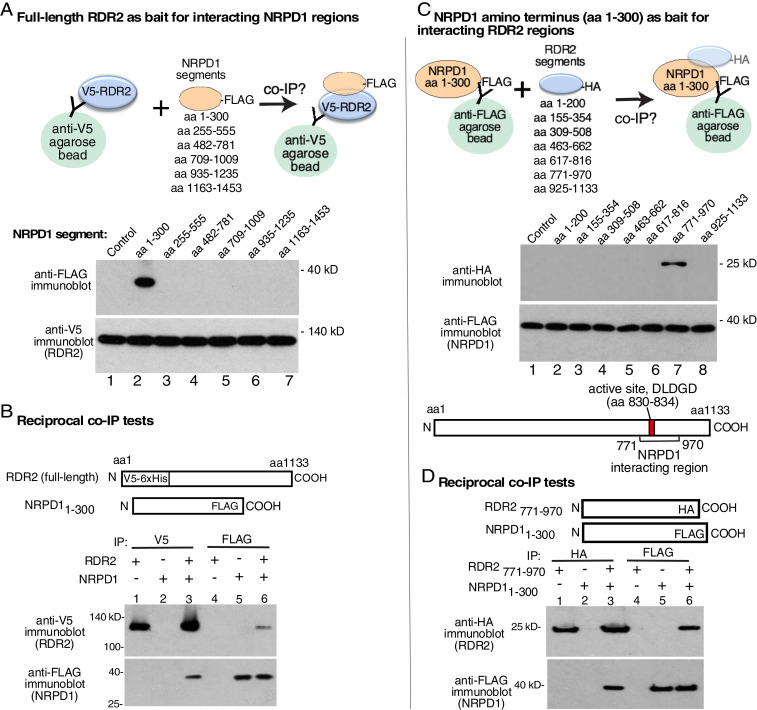
Mapping interacting subdomains of NRPD1 and RDR2. (*A*) Testing NRPD1 amino acid intervals for interaction with RDR2. Six overlapping polypeptides that collectively represent the 1,453 amino acids of full-length NRPD1 were designed, each having a C-terminal FLAG tag. The polypeptides were then expressed in vitro using a cell-free transcription-translation system (*SI Appendix*, Fig. S1*A*) and incubated with V5-tagged recombinant RDR2. Anti-V5 immunoprecipitation was then used to pull down RDR2 and any associated proteins, as depicted in the cartoon. IPed proteins were resolved by SDS-PAGE and subjected to immunoblotting using anti-FLAG and anti-V5 antibodies. Immunoblots were probed with anti-FLAG and anti-V5. The control is a mock reaction in which the bacterial transcription-translation system was not provided with an NRPD1 coding sequence to transcribe or translate prior to incubation with v5-tagged RDR2. (*B*) NRPD1_1–300_ and RDR2 co-IP. Recombinant, FLAG-tagged NRPD1_1–300_ expressed in *E. coli* (*SI Appendix*, Fig. S1*B*) was incubated with recombinant V5-tagged RDR2 (lanes 3 and 6), then IPed using either anti-FLAG or anti-V5 resin. In lanes 1, 2, 4, and 5, the individual proteins were IPed as controls. (*C*) Testing RDR2 amino acid intervals for interaction with NRPD1_1–300_. Seven overlapping polypeptides of ∼22 kDa that collectively represent the amino acid sequence of full-length RDR2 (1,133 amino acids) were designed, each having a C-terminal HA epitope tag. The recombinant RDR2 polypeptides were then expressed in vitro using a cell-free transcription-translation system (*SI Appendix*, Fig. S2*A*). The RDR2 polypeptides were then incubated with NRPD1_1–300_ followed by anti-FLAG IP to pull down NRPD1_1–300_ and any associated proteins, as depicted in the cartoon at *Top*. IPed proteins were resolved by SDS-PAGE and subjected to immunoblotting using anti-FLAG and anti-HA antibodies. Immunoblots were probed with anti-HA and anti-FLAG. The diagram at *Bottom* shows that RDR2_771–970_ includes the enzyme’s active site. The control is a mock reaction in which the bacterial transcription-translation system was not provided with an RDR2 coding sequence to transcribe or translate prior to incubation with FLAG-tagged NRPD1. (*D*) Recombinant NRPD1_1–300_ and recombinant RDR2_771–970,_ each expressed in *E. coli* (*SI Appendix*, Figs. S1*B* and S2*B*) were incubated together (lanes 3 and 6) or alone (lanes 1, 2, 4, and 5), then subjected to anti-HA or anti-FLAG IP. IPed proteins were resolved by SDS-PAGE and subjected to immunoblotting using anti-FLAG or anti-HA antibodies.

Full-length NRPD1 is 1,453 amino acids in length. To search for regions that might be sufficient to interact with RDR2, we engineered six recombinant expression vectors, each encoding ∼33-kDa portions of NRPD1 (*SI Appendix*, Fig. S1*A*) that overlap by ∼5 kDa and each having a C-terminal FLAG epitope tag. The polypeptides were generated in vitro using a bacterial transcription-translation expression system then incubated with V5-tagged recombinant RDR2 (see cartoon of Fig. 4*A*). Following anti-V5 IP, proteins were subjected to SDS-PAGE and immunoblotting, with anti-V5 antibody used to detect RDR2 and anti-FLAG antibody used to detect NRPD1 polypeptides. The polypeptide corresponding to the amino terminus of NRPD1, amino acids 1–300, co-IPed with RDR2 (Fig. 4*A*, lane 2). The partially overlapping polypeptide corresponding to amino acids 255–555 did not co-IP with RDR2 (Fig. 4*A*, lane 3), suggesting that RDR2 interaction likely occurs within NRPD1’s first 255 amino acids.

To further test the ability of the NRPD1_1–300_ polypeptide to interact with RDR2, we engineered a recombinant gene that expresses NRPD1_1–300_ in *Escherichia coli* and affinity purified the polypeptide by virtue of its C-terminal FLAG tag (*SI Appendix*, Fig. S1*B*). Following incubation with V5-tagged RDR2, reactions were IPed using anti-V5 or anti-FLAG resin. IPed proteins were then subjected to SDS-PAGE and immunoblotting, using anti-V5 antibody to detect RDR2 and anti-FLAG antibody to detect NRPD1_1–300_ (Fig. 4*B*). In each test, RDR2 and NRPD1_1–300_ co-IPed (see Fig. 4*B*, lanes 3 and 6).

### A Site Flanking the RDR2 Active Site Interacts with NRPD1.

To identify regions of RDR2 that interact with NRPD1, we engineered constructs encoding seven overlapping polypeptides of ∼25 kDa, each bearing an HA epitope tag, and expressed the polypeptides in vitro using the bacterial transcription-translation system (*SI Appendix*, Fig. S2*A*). Following incubation with FLAG-tagged NRPD1_1–300_, IP was conducted using anti-FLAG resin and affinity-captured proteins were subjected to SDS-PAGE and immunoblotting using anti-HA or anti-FLAG antibodies (Fig. 4*C*). The polypeptide corresponding to RDR2 amino acids 771–970, which includes the conserved aspartate triad of the active site, co-IPs with NRPD1_1–300_ (Fig. 4*C*, lane 7). The partially overlapping polypeptides corresponding to amino acids 617–816 or 925–1133 did not co-IP with NRPD1_1–300_. Collectively, these results suggest that RDR2 sequences between amino acids 816 and 925 can interact with NRPD1.

To confirm the ability of RDR2_771–970_ to interact with FLAG-tagged NRPD1_1–300_, we expressed RDR2_771–970_, fused to an HA epitope tag, in *E. coli* and affinity-purified the protein using anti-HA resin (*SI Appendix*, Fig. S2*B*). We then incubated the protein with FLAG-tagged NRPD1_1–300_ and performed IP using anti-FLAG or anti-HA resin. These tests showed that RDR2_771–970_ and NRPD1_1–300_ co-IP, regardless of which partner is affinity-captured (Fig. 4*D*, lanes 3 and 6). Analysis of the RDR2_771–970_ and NRPD1_1–300_ polypeptides by circular dichroism spectroscopy showed that both proteins displayed spectral dips indicative of alpha helices and/or beta sheets (*SI Appendix*, Fig. S3). This indicates that the polypeptides have secondary structure and that interactions between the polypeptides occur in the context of proteins that are at least partially folded.

### Fine Mapping Pol IV-RDR2 Interaction Sites Using Peptide Arrays.

**Fig. 5.**
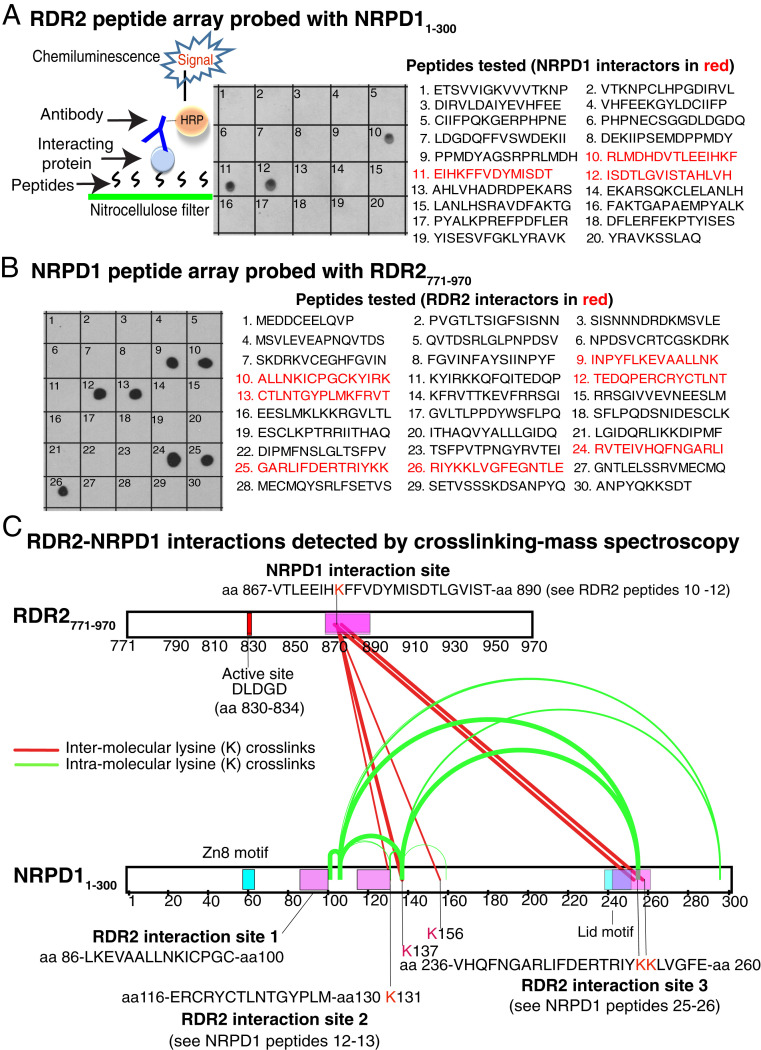
Interacting peptides of NRPD1 and RDR2. (*A*) Peptides of RDR2_771–970_ that interact with NRPD1_1–300_. Twenty overlapping 15-amino acid peptides were dot-blotted onto nitrocellulose and incubated with NRPD1_1–300_-FLAG. After washing, the filter was probed with anti-FLAG antibody conjugated to HRP. HRP activity was then detected by chemiluminescence. (*B*) Peptides of NRPD1_1–300_ that interact with RDR2_771–970_.Thirty overlapping 15-amino acid peptides were dot-blotted onto nitrocellulose and incubated with RDR2_771–970_ -HA. After washing, the filter was probed with anti-HA antibody conjugated to HRP. HRP activity was then detected by chemiluminescence. (*C*) RDR2 -NRPD1 interactions detected by cross-linking and mass spectroscopy. NRPD1_1–300_ and RDR2_771–970_ were incubated together with the chemical cross-linker BS3, subjected to SDS-PAGE to resolve cross-linked from noncross-linked polypeptides, then extracted from the gel and analyzed by mass spectroscopy. Cross-links between lysines of the two proteins are shown as red lines, with thick lines denoting the highest confidence interactions (Rank 1, see *SI Appendix*, *Methods* for details) and thin lines depicting Rank 4 interactions that involve short peptides (<5 amino acids). Cross-links between lysines of NRPD1 are shown as green arcs, with the thickest lines depicting Rank 1, medium thickness lines depicting Rank 2, and thin lines depicting Rank 4 interactions. Regions of RDR2 and NRPD1 found to interact in other assays are depicted as pink rectangles. The Zn8 and Lid elements of NRPD1 are shown as blue rectangles. The overlap between the Lid and RDR2 interaction site 3 is depicted in purple. Data are available from ProteomeXchange via the identifier PXD020170 (39).

Having identified regions of NRPD1 and RDR2 that interact, we next searched for peptides within these regions that might account for the interactions. Twenty peptides that collectively comprise the amino acids of RDR2_771–970_ and 30 peptides that comprise the NRPD1_1–300_ sequence were synthesized and arrayed by dot blotting onto nitrocellulose (Fig. 5 *A* and *B*). Peptides were typically 15 amino acids in length and overlapped their neighbors by 5 amino acids. The RDR2 peptide array was incubated with NRPD1_1–300_-FLAG, and the NRPD1 peptide array was incubated with RDR2_771–970_-HA. Filters were washed to remove unbound probe proteins and then incubated with HRP-conjugated antibodies recognizing the HA or FLAG tags of NRPD1_1–300_ or RDR2_771–970_. Filters were again washed then assayed for HRP-catalyzed chemiluminescence to screen for the presence of immobilized probe polypeptides (see cartoon of Fig. 5*A*).

Three contiguous peptides of RDR2, peptides 10–12, interact with NRPD1_1–300_ (Fig. 5*A*). If shared sequences of noninteracting, overlapping peptides 9 and 13 are excluded, the sequence ^866^DVTLEEIHKFFVDYMISDTLGVIST^890^ emerges as the candidate NRPD1 contact region. This sequence is close to the active center, beginning 32 amino acids downstream from the ^830^DLDGD^834^ motif that coordinates a magnesium ion at the site of phosphodiester bond formation (see upper diagram of Fig. 5*C*).

In the case of NRPD1, three RDR2-interacting intervals were detected, defined by peptides 9–10, 12–13, and 24–26 (Fig. 5*B*). Omitting shared amino acid sequences of noninteracting adjacent peptides, the sequences ^86^LKEVAALLNKICPGC^100^, ^116^ERCRYCTLNTGYPLM^130^, and ^236^VHQFNGARLIFDERTRIYKKLVGFE^260^ are deduced to contain RDR2 interaction motifs.

### Mapping Pol IV-RDR2 Interaction Sites by Chemical Cross-Linking and Mass Spectroscopy.

As an independent means of identifying interacting peptides of NRPD1 and RDR2, we incubated NRPD1_1–300_ and RDR2_771–970_ with the lysine cross-linker bis-sulfosuccinimidyl suberate (BS3). We then used SDS-PAGE to gel-purify cross-linked complexes, performed digestion with trypsin or chymotrypsin, and identified resulting peptides by mass spectroscopy (Fig. 5*C*). Cross-linked peptides of each protein were detected, including intraprotein and interprotein cross-links. Cross-links between RDR2 and NRPD1 occurred between lysine 874 (K874) of RDR2_771–970_ and NRPD1_1–300_ lysines K131, K137, K156, K254, and K255 (Fig. 5*C*, red lines). Importantly, lysine 874 of RDR2 is present within peptides 10 and 11 of the peptide array (Fig. 5*A*) and is the only lysine within NRPD1-interacting peptides 10–12. This region of RDR2 is depicted as a pink rectangle in the diagram of Fig. 5*C*. Likewise, two of the three RDR2-interacting regions of NRPD1 previously defined in the peptide array experiment included, or were immediately adjacent to, lysines that were cross-linked to RDR2, specifically K131, which is present in peptide 13, and K254 and K255, present within peptides 25 and 26 (Fig. 5 *C* and *B*). Moreover, cross-links formed between lysines within NRPD1_1–300_ (green arcs in Fig. 5*C*) indicate that RDR2 interacting region 3, which is seemingly far apart from interacting regions 1 and 2 in a linear depiction of the protein, must be in close proximity to these regions in the folded polypeptide because the distance between the reactive groups of the BS3 cross-linker is only 11.4 Å. Collectively, the results suggests that NRPD1_1–300_ peptides 9–10, 12–13, and 24–26 may be part of a larger RDR2-binding surface.

### Pol IV-RDR2 Interaction Sites Are Conserved in Plants.

**Fig. 6.**
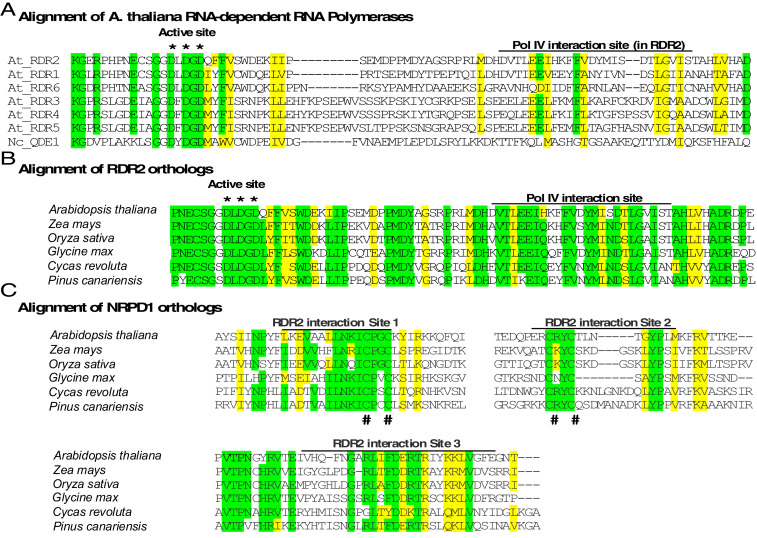
Sequence conservation at RDR2 and NRPD1 interaction sites. (*A*) Alignment of *A. thaliana* RNA-dependent RNA polymerases RDR1 through RDR6 and *N. crassa* QDE-1, in the region that includes the conserved active site aspartates (*) and Pol IV (NRPD1) interaction site. Identical amino acids are highlighted in green; similar amino acids are highlighted in yellow. The cysteines of the putative zinc coordination sites in RDR2 interactions sites 1 and 2 are denoted by (#). (*B*) Sequence conservation at the Pol IV (NRPD1) interaction site among RDR2 orthologs of *A. thaliana*, maize (*Zea mays*), rice (*Oryza sativa*), soybean (*Glycine max*), cycad (*Cycas revoluta*) and pine (*Pinus canariensis*). (*C*) Sequence conservation at the RDR2-interaction sites of NRPD1 orthologs of maize, rice, soybean, cycad, and pine.

The *A. thaliana* genome encodes six RNA-dependent RNA polymerases (RdRPs) but only RDR2 interacts with Pol IV. The NRPD1 interaction site within RDR2 is deduced to involve the sequence DVTLEEIHKFFVDYMISDTLGVIST, as discussed above. This sequence has little similarity to the corresponding sequences of RDRs 1, 3, 4, 5, or 6 (Fig. 6*A*). By contrast, the nearby active site region is highly conserved, especially between RDR2 and its closest paralogs, RDR1 and RDR6. The conservation at the active site extends to the *Neurospora crassa* RNA-dependent RNA polymerase, QDE-1 (29).

RDR2 and NRPD1 copurify in *A. thaliana*, a dicotyledonous (dicot) plant and maize, a monocotyledonous plant, whose last common ancestor existed ∼150–200 million years ago. Fig. 6*B* compares the NRPD1-interaction site of RDR2 and its orthologs in two dicots (*Arabidopsis* and *Glycine*), two monocots (*Zea* and *Oryza*), and two gymnosperms, *Pinus* and *Cycas*, who last shared a common ancestor with monocots and dicots ∼400 million years ago. Substantial sequence conservation is apparent. Likewise, alignment of the three RDR2 interaction sites of *Arabidopsis* NRPD1 with orthologs of the other species also reveals blocks of identical or similar amino acids (Fig. 6*C*). Collectively, the high degree of sequence conservation at NRPD1 and RDR2 interaction sites suggest that Pol IV-RDR2 complexes may assemble in the same way throughout the plant kingdom.

## Discussion

**Fig. 7.**
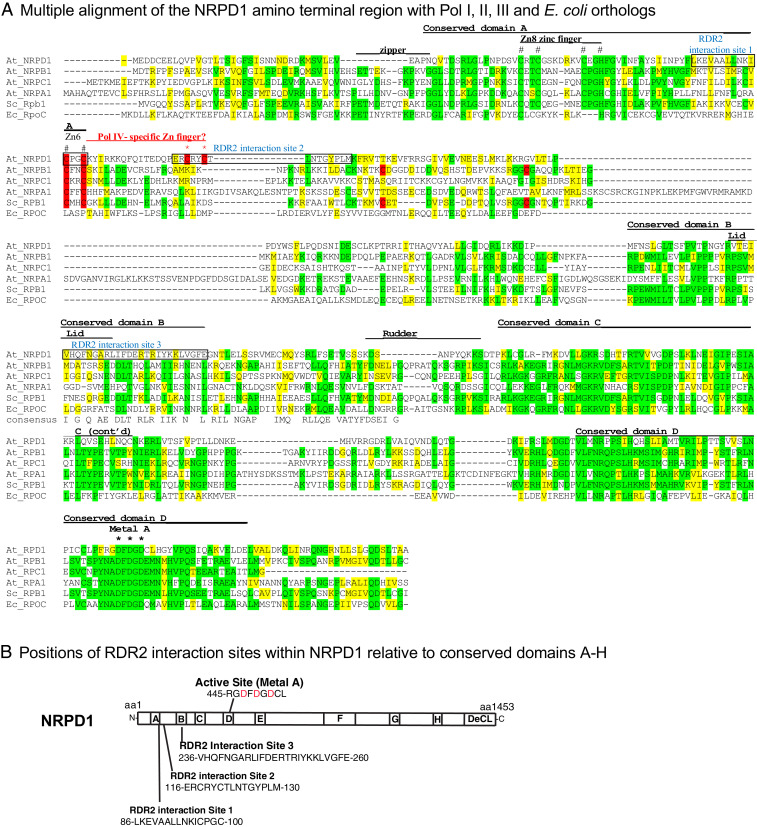
Comparison of amino terminal regions of RNA polymerase largest subunits. (*A*) Multiple alignment of NRPD1’s amino terminus with corresponding sequences of the largest subunits of *Arabidopsis* (At) Pols I (NRPA1), II (NRPB1), and III (NRPC1); budding yeast Pol II (Sc_RPB1); and *E. coli* polymerase (Ec_RpoC). Identical amino acids in three or more sequences are shaded green; similar amino acids are shaded yellow. Positions of conserved domains A–D, zipper, lid, and rudder elements are indicated based on their positions in yeast Pol II. The position of the Metal A site is also indicated, with black asterisks denoting the three magnesium-binding aspartates. Conserved Zinc coordinating amino acids of Zn8 and Zn6 are indicated with hash symbols (#). Cysteines of Zn6 are further highlighted in red, with cysteines of the Pol IV-specific CxxC, motif downstream of the conserved CxxC motif, indicated with red asterisks. RDR2 interacting regions 1, 2, and 3 of NRPD1, which overlap both Zn6 CxxC motifs and the lid element, respectively, are indicated in blue. (*B*) Relative locations of the active site and RDR2-interacting regions in a linear depiction of full-length NRPD1. Positions of domains A–H, conserved among largest subunits of all multisubunit RNA polymerases, and the C-terminal DeCL domain (also present in the Pol V largest subunit) are highlighted.

Our results indicate that Pol IV and RDR2 can form a stable complex that involves interactions between RDR2 and the largest of Pol IV’s 12 subunits. Three RDR2-interacting regions of NRPD1 are present within the first 300 amino acids of the protein. This region includes conserved domains A and B, which are two of eight domains (A–H) conserved among multisubunit RNA polymerase largest subunits, and ends just prior to the beginning of conserved domain C (see Fig. 7 for positions of these domains). These first 300 amino acids include conserved and polymerase-specific elements of the structural module known as the clamp. Structural studies of bacterial, archaeal, and eukaryotic RNA polymerases have shown that the clamp adopts different conformations, opening to allow DNA entry into the cleft where the catalytic site is located, and rotating inward to enclose the DNA-RNA hybrid that forms during transcription elongation (30–35). The clamp includes core and head subdomains, with clamp cores of eukaryotic Pols I, II, and III and archaeal RNA polymerases adopting nearly identical structures. By contrast, head subdomains are not conserved and form structures that facilitate interactions with polymerase-specific transcription factors or subunits. Smaller functional elements of the clamp include the zipper, lid, and rudder loops that differ in sequence among different polymerases but are strategically located to help define the upstream end of the DNA-RNA hybrid and separate the RNA transcript from the DNA template at the base of the RNA exit channel (30–32). In Fig. 7*A*, the locations of these elements are shown based on their positions in Pol II (30, 31).

Within NRPD1, RDR2 interaction site 1 is located within conserved domain A, just downstream of the CxxC-CxxH zinc finger known as Zn8 (in Pol II) and including the first CxxC (highlighted in red and marked by hash symbols, #, in Fig. 7*A*) of the adjacent zinc coordinating element, Zn6. These zinc binding amino acids are invariant in the largest subunits of eukaryotic RNA polymerases I, II, III, and IV (Fig. 7*A*). Genetic experiments have shown that substitutions at these invariant amino acids in Pol II confer temperature-sensitive or lethal phenotypes (36). In Pols I, II, and III, Zn8 and all preceding sequences contribute to their structurally similar clamp cores, whereas the conserved CxxC motif of Zn6 contributes to their clamp heads. Given the conservation at these sites, it is likely that the same is true for Pol IV.

In yeast Pol II, Zn6 coordination involves the highly conserved CxxC motif and two cysteines (also highlighted in red in Fig. 7*A*) located 38 and 57 amino acids downstream, respectively. However, in NRPD1 the highly conserved Zn6 CxxC motif is followed, 17–20 amino acids downstream, by a second CxxC motif (highlighted in red and marked by asterisks in Fig. 7*A*) that is not found in the largest subunits of Pols I, II, and III. This second, Pol IV-specific CxxC motif is part of RDR2-interaction site 2. Thus, RDR2 interaction sites 1 and 2 each include CxxC motifs, and both motifs are highly conserved (Fig. 6*C*). These CxxC motifs in NRPD1 have the potential to coordinate a zinc atom, thereby generating a Pol IV-specific zinc finger within the RDR2 interacting region. However, our data suggest that formation of this putative zinc finger is not critical for RDR2 docking given that peptides that include the individual CxxC motifs (peptides 10 and 12) bind RDR2 in the peptide array experiment, as did flanking peptides 9 and 13, which lacked the CxxC motifs. These results suggest that RDR2 can bind independently to either side of the putative zinc finger in a sequence-specific manner, consistent with the conservation of sequences adjacent to the CxxC motifs (Fig. 6*C*). Moreover, a tyrosine substitution at the first cysteine of the CxxC motif at the beginning of RDR2 interaction site 2 (C118Y, defining mutant allele *nrpd1-50*) did not disrupt RDR2 interaction, despite severely impairing Pol IV activity in vivo and in vitro (37). Thus, the putative zinc finger may play a role in Pol IV transcription independent of RDR2 interaction.

NRPD1 has amino acid substitutions at the position of the lid (Fig. 7*A*), which in other polymerases is implicated in helping separate the RNA transcript from the DNA template at the upstream edge of the DNA-RNA hybrid. Interestingly, the amino acids at the expected position of the lid overlap with RDR2 interaction site 3. Collectively, the evidence point to RDR2 interactions with NRPD1 in regions that help define the Pol IV catalytic center, likely positioning the enzymes to facilitate the transfer of Pol IV transcripts to RDR2 (13). Structural studies of Pol IV, RDR2, and the complex formed by both proteins will likely prove informative for further revealing the basis for Pol IV-RDR2 transcription coupling.

## Materials and Methods

### Cloning, Expression, and Purification of Active or Catalytically Dead RDR2.

An RDR2 cDNA expression construct encoding the WT protein fused to V5 epitope and 6xHis tags was described previously (10). An active site mutant (RDR2-asm) was generated by site-directed mutagenesis using primers listed in the *SI Appendix*, Table S1. The RDR2 and RDR2-asm genes were expressed in insect cells as BaculoDirect expression vectors (Thermo Fisher Scientific). Recombinant proteins were affinity-purified using nickel-nitrilotriacetic acid (Ni-NTA) agarose (Qiagen) and elution with imidazole, followed by fast protein liquid chromatography (FPLC) using Heparin-Sepharose and Superdex 200 columns. Purity was assessed by SDS-PAGE and staining with Coomassie Blue. See *SI Appendix*, *Methods* for additional details.

### Analytical Size-Exclusion Chromatography.

To estimate RDR2’s mass in solution, RDR2 and protein standards (GE Healthcare) were subjected to FPLC using a Superdex 200 10/300 GL column at 4 °C using 50 mM Hepes-KOH pH 7.5, 150 mM NaCl as the running buffer and a flow rate of 0.3 mL/min. Protein peaks were detected by UV absorbance at 280 nm, and SDS-PAGE with staining by Coomassie Blue.

### RDR2 Activity Assay.

Conversion of ssRNA into dsRNA was carried out in 50-μL reactions containing 200 ng of RDR2, 25 nM template RNA (a 37-nt RNA 5′ end-labeled using T4 kinase and gamma-[^32^P]ATP), 25 mM Hepes-KOH pH 7.5, 2 mM MgCl_2_, 0.1 mM EDTA, 0.1% Triton X-100, 20 mM ammonium acetate, 3% PEG 8000, 0.1 mM each of ATP, GTP, CTP, and UTP, and 0.8 U/μL RiboLock RNase inhibitor (Thermo Fisher Scientific). Reactions were incubated at room temperature and stopped by adding EDTA to 10 mM. For RNase treatments, 1 unit of RNase ONE (Promega) or 0.01 unit of RNase V1 (Thermo Fischer Scientific) was added to reaction products and incubated for 15 min at 37 °C. Reactions were stopped by adding SDS to 0.1% (wt/vol). Reaction products were subjected to TRIzol extraction, precipitated with ethanol, resuspended in nuclease-free water, adjusted to 1× DNA loading dye (Thermo Fisher Scientific), and subjected to nondenaturing gel electrophoresis using a 15% polyacrylamide gel and 0.5× TBE (Tris-Borate-EDTA) running buffer. The gel was transferred to blotting paper, covered with plastic wrap and exposed to X-ray film at −80 °C for 1 h.

### Reconstitution of Pol IV-RDR2 Complexes.

Pol IV, Pol V, and Pol II were affinity-purified by virtue of their FLAG-tagged NRPD1, NRPE1, or NRPB2 subunits as described previously (26). To reconstitute Pol IV-RDR2 complexes, Pol IV expressed in a *rdr2* null mutant background was immobilized on anti-FLAG M2 resin then incubated with 1 µM recombinant RDR2 for 30 min at 4 °C in 50 mM Hepes-KOH pH 7.5, 150 mM NaCl. The resin was then washed three times with 100 μL of the same buffer to remove unbound proteins then subjected to SDS-PAGE and immunoblotting using anti-FLAG-HRP (Sigma Aldrich) or antibodies recognizing native NRPD1, NRPE1, or NRPB2. Anti-V5-HRP antibody (Invitrogen) was used to identify recombinant RDR2.

### Assay for dsRNA Synthesis by the Coupled Reactions of Pol IV and RDR2.

Pol IV isolated in association with native RDR2, or Pol IV isolated from an *rdr2* null mutant (26) and reconstituted with recombinant RDR2, were tested for dsRNA synthetic ability as in Singh et al. (13). Reactions included 50 μL of anti-FLAG resin bearing immobilized Pol IV, with or without associated RDR2, in a total volume of 100 μL. Final concentrations of reaction components were 20 mM Hepes-KOH pH 7.6, 100 mM potassium acetate, 60 mM ammonium sulfate, 10 mM magnesium sulfate, 10% vol/vol glycerol, 10 μM zinc sulfate, 0.1 mM phenylmethylsulfonyl fluoride, 1 mM dithiothreitol, 0.8 U/μL Ribolock (Thermo Fisher Scientific). To specifically label RNA strands synthesized by RDR2, reactions were performed using 250 nM T-less template DNA (lacking thymidines); 1 mM each of GTP, CTP, and UTP; 40 μM ATP; and 10 μCi of [α^32^P]-ATP (3,000 Ci/mmol; PerkinElmer). Transcription reactions were incubated 1 h at room temperature on a rotating mixer, stopped by the addition of EDTA to 25 mM, and incubated at 75° C for 10 min. Transcription reactions were then passed through PERFORMA spin columns (Edge Bio) and adjusted to 0.3 M sodium acetate (pH 5.2). Fifteen micrograms of Glycoblue (Thermo Fisher Scientific) was added, and RNAs were precipitated with 3 volumes of isopropanol at −20 °C. Following centrifugation, pellets were washed with 70% ethanol and resuspended in 5 μL of water. Five microliters of 2× RNA loading dye (New England Biolabs) was added, and the samples were heated for 5 min at 75° C. RNAs were resolved on 15% polyacrylamide, 7 M urea gels. Gels were transferred to filter paper, vacuum dried, and subjected to autoradiography or phosphorimaging.

### Cloning, Expression, and Purification of NRPD1.

The NPRD1 open reading frame, fused to a C-terminal FLAG tag and codon optimized for insect cell expression, was synthesized by GenScript and cloned into the baculovirus expression vector pKL-10xHis-MBP-SED-3C. The vector was first transfected into Sf9 insect cells to produce recombinant baculovirus particles, then High Five cells for NRPD1 overexpression. The NRPD1-Maltose Binding Protein (MBP) fusion protein was affinity-purified using Amylose resin (New England Biolabs) and eluted with 20 mM maltose. The fusion protein was next subjected to anti-FLAG affinity purification, with in-column Prescission Protease digestion used to cleave the linker between MBP and NRPD1. Following extensive washing to remove free MBP, NRPD1 was eluted using an excess of FLAG peptide. See *SI Appendix*, *Methods* for additional details.

### Cloning, Overexpression, and Purification of NRPD1_1–300_ and RDR2_771–970_.

NRPD1 amino acids 1–300 fused to His and FLAG tags, and RDR2 amino acids 771–971 fused to His and HA tags, were expressed in *E. coli* using pET28 vectors. The proteins were then purified using nickel-NTA column chromatography and elution with imidazole. Purity was assessed by SDS-PAGE and immunoblotting using anti-FLAG-HRP or anti-HA-HRP antibodies. See *SI Appendix*, *Methods* for additional details.

### Coimmunoprecipitation Experiments.

Recombinant RDR2 and NRPD1 (0.25 µM each) were mixed and incubated for 30 min in 50 mM Hepes-KOH pH 7.5, 150 mM NaCl (at room temperature, in a volume of 100 µL). Fifty-microliter aliquots were then mixed with 50 µL of anti-FLAG or anti-V5 antibody resin. After 30 min at room temperature, the resins were washed twice with 200 µL of 50 mM Hepes-KOH (pH 7.5), 150 mM NaCl. Proteins were eluted by the addition of 6× SDS loading dye, and heating at 95 °C for 2 min, then subjected to SDS-PAGE. Immunoblot analysis was used to detect the proteins using anti-FLAG-HRP to detect NRPD1 or anti-V5-HRP to detect RDR2 antibodies. The same methods were used to test for co-IP of full-length RDR2 with NRPD1_1–300_ or for co-IP of NRPD1_1–300_ with RDR2_771–970_.

### Yeast Two-Hybrid Interaction Assay.

*Saccharomyces cerevisiae* strain MaV203 was transformed according to the Matchmaker protocol (Thermo Fisher) with plasmids expressing NRPD1 or RDR2 fused to the GAL4 activation domain or GAL4 DNA-binding domain. Transformants were selected on SC-Leu-Trp plates for 3 d at 30 °C. To test for interactions, each strain was replica plated onto SC,-Trp,-Leu and SC,-Trp,-Leu,-His, +3AT agar. Growth after 3 d at 30 °C was then assessed.

### Expression of NRPD1 and RDR2 Polypeptides.

Subregions (20–33 kDa) of NRPD1 and RDR2 polypeptides were expressed in vitro using a PURExpress In Vitro Protein Synthesis Kit (New England Biolabs). Briefly, the desired intervals of the synthetic genes were amplified using primers listed in *SI Appendix*, Table S1 and then used as templates for in vitro transcription-translation reactions.

### Peptide Array Protein Interaction Assays.

Custom peptide libraries corresponding to NRPD1 amino acids 1–300 or RDR2 amino acids 771–971 were obtained from Genscript and dot-blotted (10 ng) onto nitrocellulose. Filters were submerged in 5% (wt/vol) nonfat dry milk in 1× TBST (Tris buffered saline, 0.1% Tween 20), 30 min at room temperature to block free protein binding sites. The blocking solution was then discarded. NRPD1 peptide arrays were then probed with ∼200 ng of RDR2_771–971_-HA in 8 mL of 50 mM Hepes-KOH pH 7.5, 100 mM NaCl at room temperature for 1 h. The RDR2 peptide array was probed with ∼200 ng of NRPD1_1–300_-FLAG. Filters were then washed twice with 8 mL of 1× TBST, then incubated with anti-FLAG-HRP or anti-HA-HRP in 8 mL of 1× TBST at room temperature for 1 h. The filters were washed twice with 8 mL of 1× TBST, then developed using a Pierce Enhanced Chemiluminescence Western Blotting Kit (Thermo Fisher Scientific).

### Cross-Linking-Mass Spectroscopy.

Purified RDR2_771–970_ and NRPD1_1–300_ proteins were preincubated then cross-linked using 0.1 mM Bissulfosuccinimidyl suberate (BS3). Gel-purified cross-linked protein complexes were reduced using 10 mM TCEP (Tris(2-carboxyethyl)phosphine), alkylated with 20 mM iodoacetamide, and digested for 16 h with Trypsin or Chymotrypsin, at two concentrations (0.1 mM and 0.5 mM) for each enzyme. Resulting peptides were resolved by HPLC using a C18 column and an acetonitrile gradient and subjected to electrospray ionization and analyzed using an Orbitrap Fusion Lumos mass spectrometer. Details are provided in *SI Appendix*. Resulting data have been deposited at the ProteomeXchange Consortium via the PRIDE partner repository (38) with the dataset identifier PXD020170.

### Sequence Alignments.

Amino acid sequence alignments were performed using CLUSTAL W. Conserved sequences were highlighted using BOXSHADE V3.31.

## Supplementary Material

Supplementary File

## Data Availability

Mass spectroscopy data have been deposited in ProteomeXchange Consortium PRIDE partner repository (dataset identifier PXD020170).
